# Transcatheter Tricuspid Valve Replacement for Residual Tricuspid Regurgitation After TEER

**DOI:** 10.1002/ccd.70797

**Published:** 2026-08-03

**Authors:** Fabian Voß, Philipp Mourikis, Fabian Nienhaus, Jafer Haschemi, Alexander Hoss, Tobias Zeus, Hanna Kowalski, Maximilian Spieker, Christian Jung, Patrick Horn, Hug Aubin, Artur Lichtenberg, Richard Truse, Malte Kelm, Amin Polzin

**Affiliations:** ^1^ Department of Cardiology, Pulmonology and Vascular Medicine, Medical Faculty Heinrich‐Heine University Düsseldorf Düsseldorf Germany; ^2^ Department of Cardiac Surgery, Medical Faculty Heinrich‐Heine University Düsseldorf Germany; ^3^ Department of Anesthesiology, Medical Faculty Heinrich‐Heine University Düsseldorf Germany; ^4^ CARID (Cardiovascular Research Institute Düsseldorf) Düsseldorf Germany

**Keywords:** clip excision, teer, tricuspid regurgitation, valve replacement

## Abstract

**Background:**

Residual or recurrent tricuspid regurgitation (TR) after prior transcatheter tricuspid edge‐to‐edge (T‐TEER) repair occurs in about 12% of patients within 1 year. Since surgical tricuspid valve repair or replacement is of high risk, treatment options remain scarce. Data regarding the feasibility, safety, and treatment strategies using transcatheter valve replacement (TTVR) after T‐TEER are sparse.

**Aims:**

Here, we aimed to summarize our early experience in TTVR after failed T‐TEER, analyze its feasibility and summarize the results in a distinct algorithm.

**Methods:**

Patients undergoing TTVR (*n* = 21) at the University Hospital of Duesseldorf were prospectively analyzed and stratified according prior T‐TEER procedures with implanted devices. Groups were compared in terms of procedural success, periprocedural complications, and patient outcomes. Individual treatment decisions for patients after T‐TEER were analyzed.

**Results:**

Five patients had residual/recurrent severe to torrential TR and underwent TTVR after T‐TEER, whereas 16 patients were primarily referred for TTVR. Preprocedural wire‐based intentional clip excision (WICE) was performed in 60% of these patients. Patients did not differ regarding procedural success (100% in both groups, *p* = 0.99) and periprocedural complications (0 [TTVR after TEER] vs. 5.6% [primary TTVR], *p* = 0.99 for the combined endpoint of in‐hospital death, stroke, myocardial infarction, re‐intervention or surgery, and major bleeding). A pacemaker was required in one patient of each group, *p* = 0.4). Procedural times were higher among patients with prior T‐TEER. Treatment decisions for patients after T‐TEER were integrated into a treatment algorithm.

**Conclusion:**

The valve after clip concept is feasible and the proposed algorithm may support procedural planning.

**Abbreviations**
BMI: body mass index
CAD: coronary arterty disease
COPD: chronis obstructive pulmonary disease
dpmean: transvalvular gradient
GFR: flomerular filtration rate
HFH: heartfailure hospitalizsation
ICU: intensive care unit
LVEF: left ventricular ejection fraction
pVR: primary valve replacemente
SLDA: single leaflet detatcchment
TR: tricuspid regurgitation
TTVR: tricuspid transcatheter valve replacement
VRaR: valve replacement after repair
WICE: wire‐based intentional clip excision
(T)‐TEER: (tricuspid) transcatheter edge‐to‐edge repair

## 1 Introduction

Tricuspid regurgitation (TR) is associated with high morbidity, severe impairment of the patients' quality of life and an increased risk of death [1]. Transcatheter therapies for TR, such as tricuspid transcatheter edge‐to‐edge repair (T‐TEER), have emerged in recent years. Although recent trials did not result in an improved survival after tricuspid valve repair or replacement, a substantial improvement in patients' quality of life was evident [2, 3, 4]. Still, residual or recurrent TR, with a grading of severe to torrential, occurs in about 12% of patients at 1‐year follow‐up [2]. Transcatheter valve replacement (TTVR) may facilitate a treatment option for these patients, who are often of prohibitive surgical risk [5]. However, implanted T‐TEER‐devices may impede TTVR depending on their position within the valve. Therefore, distinct strategies are needed to perform TTVR within this cohort. Nevertheless, until now, there is only anecdotal evidence from case reports to describe the feasibility and concrete strategies to perform TTVR after T‐TEER [6, 7, 8]. Therefore, we analyzed our experience in TTVR after failed T‐TEER regarding safety and feasibility as compared to a population undergoing TTVR without prior interventions. These initial individual treatment decisions were summarized into a distinct decision algorithm.

## 2 Methods

### 2.1 Study Population

Patients undergoing TTVR between May 2024 and April 2025 at the University Hospital Duesseldorf were prospectively analyzed. Data were collected from the hospital's medical information system. All treatment decisions were based on decisions by the local heart team. All patients signed informed consent to participate in our registry, which was approved by the local ethics committee (No.: 2019‐558).

Patients were grouped into two groups, routine TTVR without prior T‐TEER attempt (primary valve replacement [pVR]) and patients undergoing TTVR after prior T‐TEER with at least one TEER device in place (valve replacement after repair [VRaR]).

Afterwards, these groups were compared regarding procedural success, procedure times, and periprocedural safety outcomes (in‐hospital mortality, stroke, bleeding [≥ TVARC Type 2], major vascular complications, need for surgical intervention or re‐intervention, cardiac complications [pericardial effusion, myocardial infarction, cardiac structure perforation], device‐related complications [dislodgement, dysfunction, erosion, endocarditis, others], acute kidney injury, new‐onset atrial fibrillation/flutter, pacemaker indication within 30 days, venous thromboembolic complications) according to the TVARC definitions [9, 10].

### 2.2 Transcatheter Tricuspid Valve Replacement

Primary TTVR was performed using the EVOQUE‐System (Edwards Lifesience, Irvine, California, USA) as described before [11]. The valve size was chosen according to prior CT measurements and the manufacturer's recommendations regarding annular and ventricular oversizing.

Among patients with prior T‐TEER attempts and implanted devices within the valve, decisions regarding the need of a wire‐based intentional clip excision (WICE)‐procedure and eventual up‐sizing of the valve prosthesis were made on an individual basis. The WICE‐procedure was performed as described before [8].

### 2.3 Echocardiographic Analysis

Echocardiographic analyses were performed within the local echo laboratory by experienced investigators, blinded to the current analysis, using commercially available echo machines (GE Vivid E90 [Chicago, Illinois, United States]). All echocardiographic data were obtained in digital form and stored on a workstation for offline analysis (IntelliSpace Cardiovascular, Version 3.2, Philips). Assessment of TR, left‐ and right ventricular volumes, and function were performed according to current recommendations [12, 13, 14, 15]. The GLIDE‐Score and its parameters were assessed as described in detail before [16].

### 2.4 Statistical Analysis

Percentages were reported to describe categorical variables and median with 95% confidence intervals were reported for continuous variables. Normality distribution of continuous variables was assessed with the Shapiro−Wilk test. Comparison between two groups were performed using the Fisher's exact test for categorical variables. Differences in continuous variables between two groups were compared for significance with a two‐tailed unpaired *t*‐test. For all analyses, *p* values of < 0.05 were considered statistically significant. All analyses were performed using GraphPad Prism (Version 9; Graphpad Software, San Diego, California, USA).

## 3 Results

Overall, 21 patients were included of whom 16 were primarily treated by TTVR, whereas five underwent TTVR after prior T‐TEER. Median age was 78 [73; 83] years and 33% were male with no differences between the groups. Overall, 90% suffered from NYHA class III or IV.

Groups did not differ regarding body‐mass‐index (BMI), prior heart‐failure hospitalizations (HFH), diagnosis of coronary artery disease (CAD), or chronic obstructive pulmonary disease (COPD) as shown in Table 1. Glomerular filtration rate (GFR) and preprocedural NT‐proBNP did not differ between the groups, but patients planned for VRpR were on higher diuretic dosages when compare to pVR patients (Table 1).

**Table 1:** Baseline characteristics in patients with primary valve replacement (pVR) compared to valve replacement after repair using TEER (VRpR).

	pVR (*n* = 16)	VRpR (*n* = 5)	*p* value
Age [years]	76 [69; 83]	83 [68; 85]	0.67
Sex [% male]	37.5	20	0.62
BMI [kg/m^2^]	26.5 [22; 29]	26 [23; 41]	0.98
NYHA III/IV [%]	87.5	100	0.99
HFH [%]	43.8	80	0.31
CAD [%]	56.3	80	0.61
COPD [%]	25	0	0.53
GFR [mL/min]	51.5 [36; 61]	43 [17; 67]	0.5
NT‐proBNP [ng/L]	2691 [761; 7431]	1415 [478; 1694]	0.41
diuretics [mg furosemid]	80 [40; 160]	160 [120; 400]	**0.03**
TAPSE [mm]	16 [14; 18]	18 [13; 20]	0.78
RV width [mm]	42 [35; 50]	47 [31; 51]	0.91
LVEF [%]	55 [37; 66]	53 [43; 74]	0.58
pasys [mmHg]	42.5 [27; 51]	33.6 [18; 39]	0.11
Cardiac index [L/min/m^2^]	2.5 [1.8; 4]	2.2 [2; 2.2]	0.55
Glide score [points]	3 [2; 4]	3 [3; 4]	0.89
Septolateral gap with > 5 mm [%]	56.2	60	0.99
Non‐anterosptal/centraljet location [%]	87.5	100	0.99
Limited image quality [%]	37.5	60	0.61
High chordal structure density	31.3	0	0.28
Star‐shaped en face morphology	87.5	100	0.99

*Note:* Data is displayed as median with 95% confidence interval. Bold printed *p*‐values indicate a significance with *p* < 0.05.

Abbreviations: CAD, coronary artery disease; COPD, chronic obstructive pulmonary disease; GFR, glomerular filtration rate; HFH, heart failure hospitalization; LVEF, left ventricular ejection fraction; pasys, systolic pulmonary artery pressure; RV, right ventricular; TAPSE, tricuspid annular plane systolic excursion.

Right ventricular function, as assessed by TAPSE, left ventricular function, systolic pulmonary artery pressures, and cardiac index did not differ between the groups (Table 1).

TR severity prior to treatment was equal in both groups (Figure 1, *p* = 0.23, respectively) with most patients suffering from massive to torrential TR (≥ 80%).

**Figure 1:**
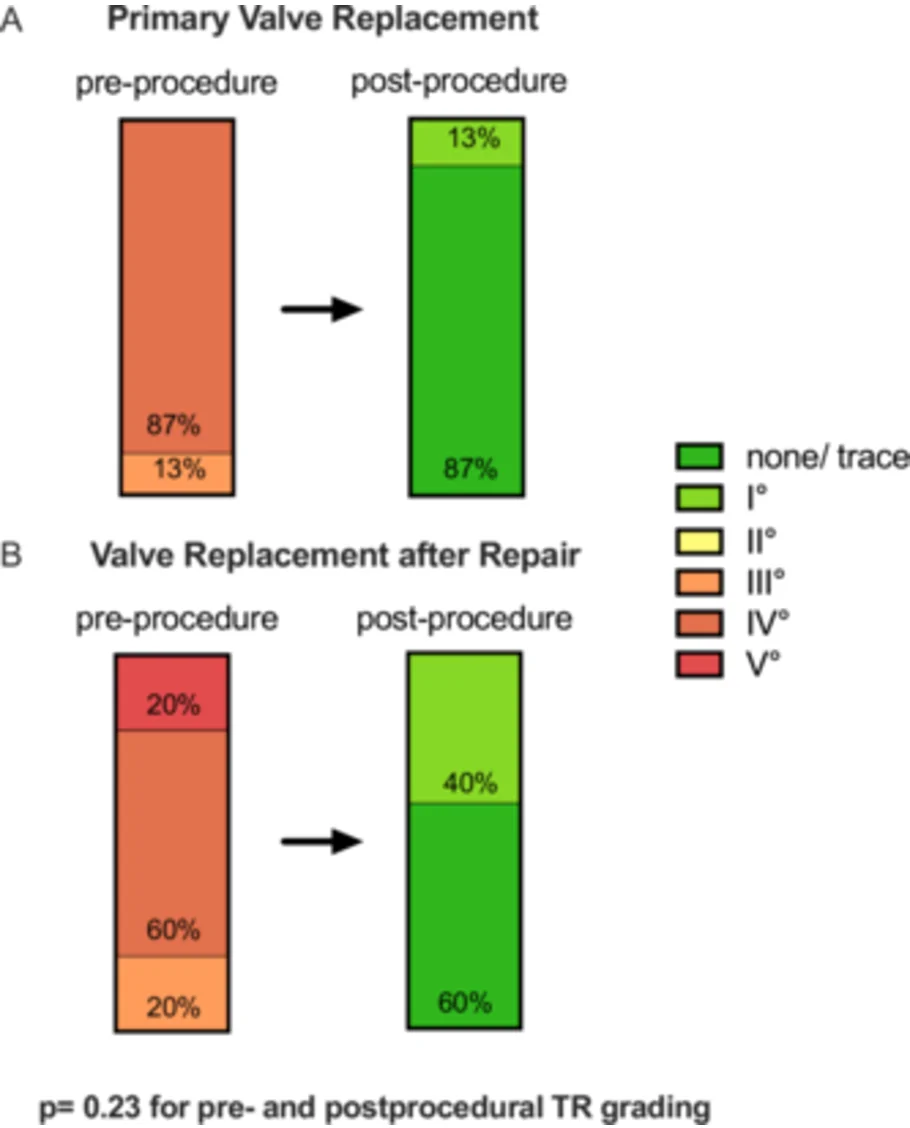
TR grading before and after TTVR in patients with primary valve replacement (A) and patients with valve replacement after TEER (B). Pre‐ and postprocedural TR did not differ between the groups (*p* = 0.23, Fisher's exact test). [Color figure can be viewed at wileyonlinelibrary.com]

Within the VRpR group five different constellations were treated as shown in Figure 2. The most common position of TEER devices was an attached device within the anterior‐septal commissure without evidence of a relevant second vale orifice (Figure 2A–C,E). An additional device with prior single leaflet detachment (SLDA) was present in one case (Figure 2B) and intentional SLDA using the WICE procedure was performed in three cases (Figure 2C–E). Two of these devices were attached to the anterior‐ and septal leaflet (Figure 2D,E), whereas one was placed between the posterior and septal leaflet (Figure 2E). The intentional SLDA has been performed to detach the anterior (Figure 2C,D) or the posterior (Figure 2E) leaflet. Accepted distances of TEER devices to proceed with TTVR without prior intentional SLDA ranged from 7.8 to 13.3 mm, each with a relative distance of less than one‐third of the total valve anteroposterior diameter. Exact T‐TEER device constellations are presented in Figure 2.

**Figure 2:**
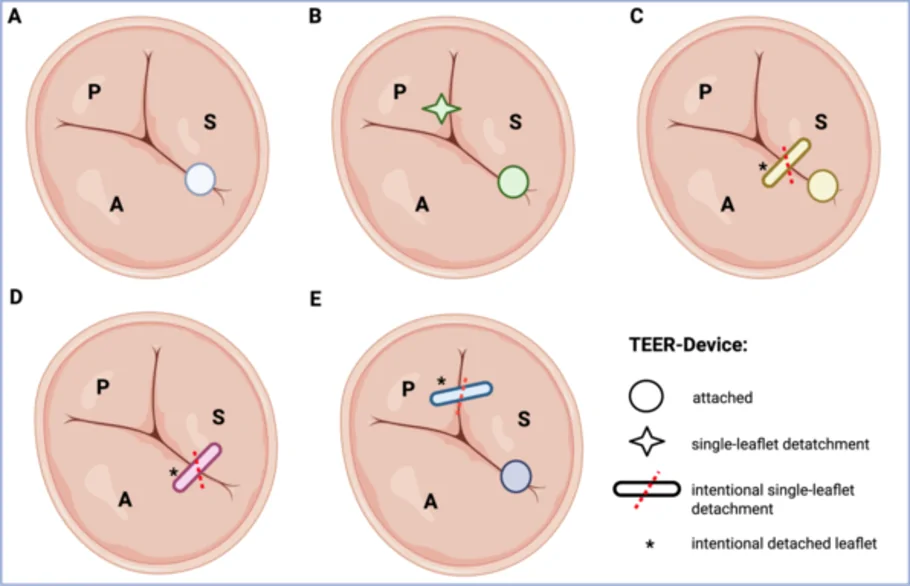
Different TEER device scenarios treated with subsequent TTVR (A−E). In three cases, a preceding WICE procedure was necessary (C−E) prior to TTVR. A, anterior leaflet; P, posterior leaflet; S, septal leaflet. Created with biorender.com. [Color figure can be viewed at wileyonlinelibrary.com]

Apart from a significantly extended procedural time within the VRaR group, mainly caused by the need for an additive WICE procedure, procedural characteristics did not differ between the groups (Table 2). Upsizing of the valve prostheses after a successful WICE procedure was performed in one case. TR was reduced to a grading of mild or less in all cases (Figure 1B), and postprocedural TR grades did not differ between the groups (*p* = 0.23).

**Table 2:** Procedural characteristics and periprocedural complications during the index hospitalization in patients with primary valve replacement (pVR) compared to valve replacement after repair using TEER (VRpR).

	pVR (*n* = 16)	VRpR (*n* = 5)	*p* value
Procedural time [min]	60 [55; 68]	120 [60; 180]	**0.01**
Procedural success [%]	100	100	0.99
Duration in hospital [days]	6 [3; 7]	5 [3; 6]	0.37
Duration on ICU [days]	1 [1; 1]	1 [1; 2]	0.99
Single/dual platelet inhibition [%]	0	25	0.53
DOAC [%]	100	81.3	0.55
Warfarin [%]	0	12.5	0.99
In‐hospital mortality [%]	0	0	0.99
Stroke [%]	0	0	0.99
Bleeding ≥ TVAR 2 [%]	6.3	0	0.99
Major vascular complications [%]	6.3	0	0.99
Re‐intervention/surgery [%]	0	0	0.99
Acute kidney injury [%]	12.5	0	0.99
Pacemaker indication	6.3	20	0.4
Mean gradient [mmHg]	3 [2.3; 4]	3.4 [3; 4.8]	0.32
*Cardiac complications*			
Pericardial effusion [%]	0	0	0.99
Myocardial infarction [%]	0	0	0.99
Cardiac structure perforation [%]	0	0	0.99
*Device‐related complications*			
Device dislodgement [%]	0	0	0.99
Device dysfunction [%]	0	0	0.99
Erosion [%]	0	0	0.99
Endocarditis [%]	0	0	0.99
Others [%]	0	0	0.99

*Note:* Data is displayed as median with 95% confidence interval. Bold printed *p*‐values indicate a significance with *p* < 0.05.

Abbreviation: ICU, intensive care unit.

Tricuspid transvalvular gradient (dpmean) did not differ between the groups (Table 2), but increased within all patients from 1 [0.9; 1.3] to 2 [1.4; 3.1] mmHg. The increase in dpmean did not differ between the groups (2 [1.4; 3.1] vs. 1.7 [−0.3; 3.5], *p* = 0.56).

Durations of both hospital stay and intensive‐care‐unit (ICU) stay were constant between the groups. In‐hospital mortality, stroke, myocardial infarction, pericardial effusion, re‐intervention, or surgery were not observed within the entire cohort (Table 2). In 6.3% of cases (*n* = 1), access‐related bleeding (rated as major or more) was observed in the pVR group. A de novo pacemaker indication was evident in one case per group (20% vs. 6.3%, *p* = 0.4, Table 2).

## 4 Discussion

Our data suggest that TTVR after failed T‐TEER is feasible in many scenarios (Figure 2) with equal reduction in TR as patients without prior T‐TEER (Figure 1). Additionally, these data do not suggest any safety concerns for TTVR after prior T‐TEER when procedural planning is performed carefully. Nevertheless, because of the limited sample size and the observational character of our analyses, conclusions regarding the procedural safety need to be done carefully until larger cohorts to confirm our findings are available. Preprocedural preparation and periprocedural adjustment of prosthesis size are needed in some cases as summarized in Figure 3. We therefore suggest a stepwise approach.

**Figure 3:**
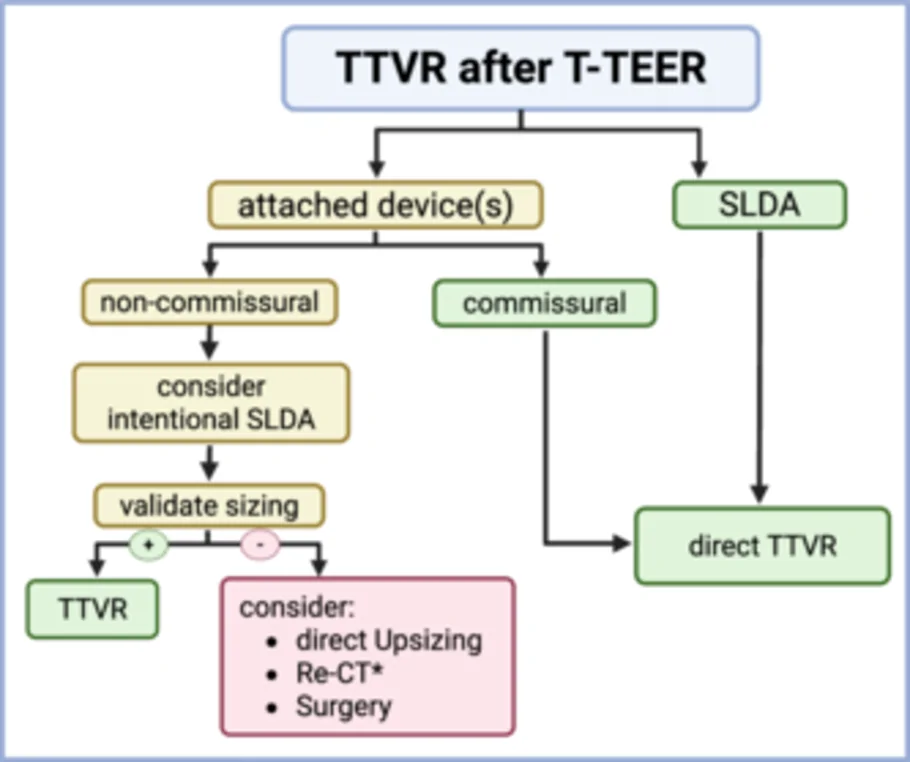
Decision tree for preparation of patients undergoing TTVR after T‐TEER. *Especially in cases of limited echocardiographic image quality and borderline measurements. Created with biorender.com. [Color figure can be viewed at wileyonlinelibrary.com]

In scenarios with T‐TEER devices located within the commissure(s) and without a second valve orifice, the TTVR might be performed as described before. If devices are attached more central, especially if a second valve orifice is present, intentional SLDA using the WICE procedure is a possible option. This procedure may prevent (a) valve dislocation caused by annulus widening after TTVR, (b) relevant perivalvular leakage (PVL), and (c) relevant concurrent flow with consecutive risk of valve thrombosis. After successful detachment of TEER devices, annulus sizing should be validated before proceeding to TTVR as described elsewhere [12]. In cases of barely oversizing of the planned TTVR prosthesis, the lost annuloplasty effect by the removal of a TEER device may lead to a relevant expansion of the TV annulus and consequently to a mismatch with the initially planned prosthesis.

In these cases, one might consider immediate up‐sizing according to echocardiographic measurements or plan a staged procedure with prior renewed CT sizing, especially in cases of limited echocardiographic image quality or borderline measurements. If the measures already exceed the limits of the available prosthesis, consider surgery or T‐TEER‐based annuloplasty as a bail‐out [17]. Since overall experience with these cases is still limited, exact limits and recommendations for upsizing or indication for renewed CT‐sizing cannot be given.

The following distinct topics need to be answered in future research and should be considered carefully during procedural planning:

1. The maximum number of implanted TEER devices which is feasible for subsequent TTVR has not been described yet. To our knowledge, patients with two implanted devices have been treated successfully [6], but nothing is known about patients with three or more devices.
2. The maximal acceptable orifice between a completely attached device and the commissure has not been defined. Double orifices of relevant size pose the risk of either concurrent flow and consecutive hemolysis and/or valve thrombosis, relevant PVL, or subsequent unintended SLDA with the risk of valve dislocation.
3. When using the WICE procedure, it needs to be considered on which leaflet the SLDA should be intended and how much leaflet is needed for successful TTVR. Additionally, the extent of a triggered flail leaflet after WICE procedures is only barely to predict and poses the risk to impede subsequent TTVR.

Importantly, although the existing trials have not been powered for these endpoints [2, 3, 4], isolated TV repair or replacement did not improve survival or frequency of heart failure hospitalizations in randomized controlled trials yet. On the other hand, isolated T‐TEER and TTVR are considered safe, leading to profound improvements in TR severity and, more importantly, the patients' quality of life [2, 3, 4]. Therefore, these complex procedures should be reserved for individuals with high symptom burden and with prohibitive risk for surgery, as suggested by local heart teams, until further trials are conducted.

Nevertheless, in addition to the limitation caused by the small sample size, the single‐center character of our cohort and the lack of randomized‐controlled trials in this field, no data concerning the long‐term durability of TTVR in general and especially after failed T‐TEER are available. Furthermore, no data regarding the effect of TEER devices on prosthesis geometry and its consequences regarding the incidence of valve thrombosis and Hypoattenuated Leaflet Thickening (HALT) is available so far.

Overall, in our very limited cohort, TTVR was feasible in patients with prior T‐TEER and residual or recurrent TR. TR was reduced to a grade of ≤ 1 in all patients without evidence of elevated gradients, relevant PVL, or valve dislocation. Furthermore, the WICE procedure facilitates the opportunity for TTVR after T‐TEER in individuals with mid‐valvular attached devices. These approaches remain individual treatment decisions since larger experience and randomized clinical trials are needed to conclude the safety and efficacy of these methods.

## Conflicts of Interest

A.P. declares the following conflicts of interest: Speakers' honoraria: Abbott, Abiomed, Amarin, AstraZeneca, Bayer, BMS, CorviaMedical, Daiichi Sankyo, Edwards, Medtronic, Occlutech, Pfizer, Philips. Advisory Board: Bayer, BMS, Boehringer Ingelheim, Pfizer, Sanofi. Clinical trials: Abbott, Abiomed, Bayer, BMS, Boston Scientific, CorviaMedical, Daiichi Sankyo, Medtronic, Novartis, Occlutech, Pfizer, Philips. Research grants: Abiomed, DFG, DZHK, DAAD, BMBF. The other authors declare no conflicts of interest.

## Data Availability

The data that support the findings of this study are available on request from the corresponding author. The data are not publicly available due to privacy or ethical restrictions.
